# Paternally inherited *ABCC8* mutation causing diffuse congenital hyperinsulinism

**DOI:** 10.1530/EDM-13-0041

**Published:** 2013-11-08

**Authors:** Suresh Chandran, Fabian Yap Kok Peng, Victor Samuel Rajadurai, Yap Te Lu, Kenneth T E Chang, S E Flanagan, S Ellard, Khalid Hussain

**Affiliations:** 1 Department of NeonatologyKK Women's and Children's Hospital100 Bukit Timah Road, Singapore, 229899Singapore; 2 Department of Paediatric EndocrinologyKK Women's and Children's Hospital100 Bukit Timah Road, Singapore, 229899Singapore; 3 Department of Paediatric SurgeryKK Women's and Children's Hospital100 Bukit Timah Road, Singapore, 229899Singapore; 4 Department of Children's PathologyKK Women's and Children's Hospital100 Bukit Timah Road, Singapore, 229899Singapore; 5 Institute of Biomedical and Clinical Science, University of Exeter Medical SchoolExeter, EX2 5DWUK; 6 Department of Paediatric EndocrinologyGreat Ormond Street Hospital for Children NHS TrustLondon, WC1N 3JHUK

## Abstract

Background: Congenital hyperinsulinism (CHI) is a rare genetic disorder characterised by inappropriate insulin secretion in the face of severe hypoglycaemia. There are two histological subtypes of CHI namely diffuse and focal. Diffuse CHI is most common due to recessive mutations in *ABCC8/KCNJ11* (which encode the SUR/KIR6.2 components of the pancreatic β-cell K_ATP_ channel) whereas focal CHI is due to a paternally inherited *ABCC8/KCNJ11* mutation and somatic loss of heterozygosity for the 11p allele inside the focal lesion. Fluorine-18-l-dihydroxyphenylalanine positron emission tomography/computed tomography (^18^F-DOPA-PET/CT) is used in the pre-operative localisation of focal lesions prior to surgery. Diffuse CHI if medically unresponsive will require a near total pancreatectomy whereas focal CHI will only require a limited lesionectomy, thus curing the patient from the hypoglycaemia.

Aims: To report the first case of genetically confirmed CHI in Singapore from a heterozygous paternally inherited *ABCC8* mutation.

Methods/Results: A term male infant presented with severe hyperinsulinaemic hypoglycaemia (HH) after birth and failed medical treatment with diazoxide and octreotide. Genetic testing (paternally inherited mutation in *ABCC8*/p.D1472N) suggested focal disease, but due to the unavailability of ^18^F-DOPA-PET/CT to confirm focal disease, a partial pancreatectomy was performed. Interestingly, histology of the resected pancreatic tissue showed changes typical of diffuse disease.

Conclusion: Heterozygous paternally inherited *ABCC8/KCNJ11* mutations can lead to diffuse or focal CHI.

**Learning points:**

- HH is a cause of severe hypoglycaemia in the newborn period.
- Paternal mutations in *ABCC8/KCNJ11* can lead to diffuse or focal disease.
- ^18^F-DOPA-PET/CT scan is the current imaging of choice for localising focal lesions.
- Gallium-68 tetra-aza-cyclododecane-*N* *N*′*N*″*N*-‴-tetra-acetate octreotate PET scan is not a useful imaging tool for localising focal lesions.
- The molecular mechanism by which a heterozygous *ABCC8* mutation leads to diffuse disease is currently unclear.
- Focal lesions are curable by lesionectomy and so genetic studies in patients with HH must be followed by imaging using ^18^F-DOPA-PET/CT scan.

## Background

Congenital hyperinsulinism (CHI) leads to severe hyperinsulinaemic hypoglycaemia (HH) in the neonatal, infancy and childhood periods. The genetic basis of CHI is beginning to be understood. Genetic studies are indicated in infants with persistent hypoglycaemia, hypoketonaemia and low fatty acid levels while requiring a glucose infusion rate (GIR) of more than 8 mg/kg per min to maintain euglycaemia (3.5–6.5 mmol/l). Most of the CHI cases are due to recessive or dominant mutations of *ABCC8/KCNJ11* genes. The majority of the recessive mutations are resistant to medical treatment. Dominant forms are characterised by the various presentations and treatment responses. Initial management is aimed at correction of hypoglycaemia to prevent long-term neuro-disability. The response to diazoxide therapy is the key to managing CHI. Diazoxide unresponsiveness is considered as an indication for a rapid mutational analysis of *ABCC8/KCNJ11* genes. Fluorine-18-l-dihydroxyphenylalanine positron emission tomography/computed tomography (^18^F-DOPA-PET/CT) scan is recommended if the genetic studies are suggestive of focal disease, which can then be cured by laparoscopic excision. We present the first case of proven CHI in Singapore in whom genetic studies suggested a focal lesion. ^18^F-DOPA-PET/CT scan is not available locally and the imaging using Gallium-68 tetra-aza-cyclododecane-*N* *N*′*N*″*N*-‴-tetra-acetate octreotate positron emission tomography (^68^G–DOTATATE-PET) and high-resolution ultrasound scan were non-conclusive. The infant underwent partial pancreatectomy with no improvement in the hypoglycaemia. Histopathology of the excised pancreas confirmed diffuse disease and the patient was subjected to near total pancreatectomy, with improvement of the HH. ^18^F-DOPA-PET/CT scanning facilities are only available in some centres around the world, thus making it difficult to identify all children with focal lesions.

## Case presentation

**Figure 1:**
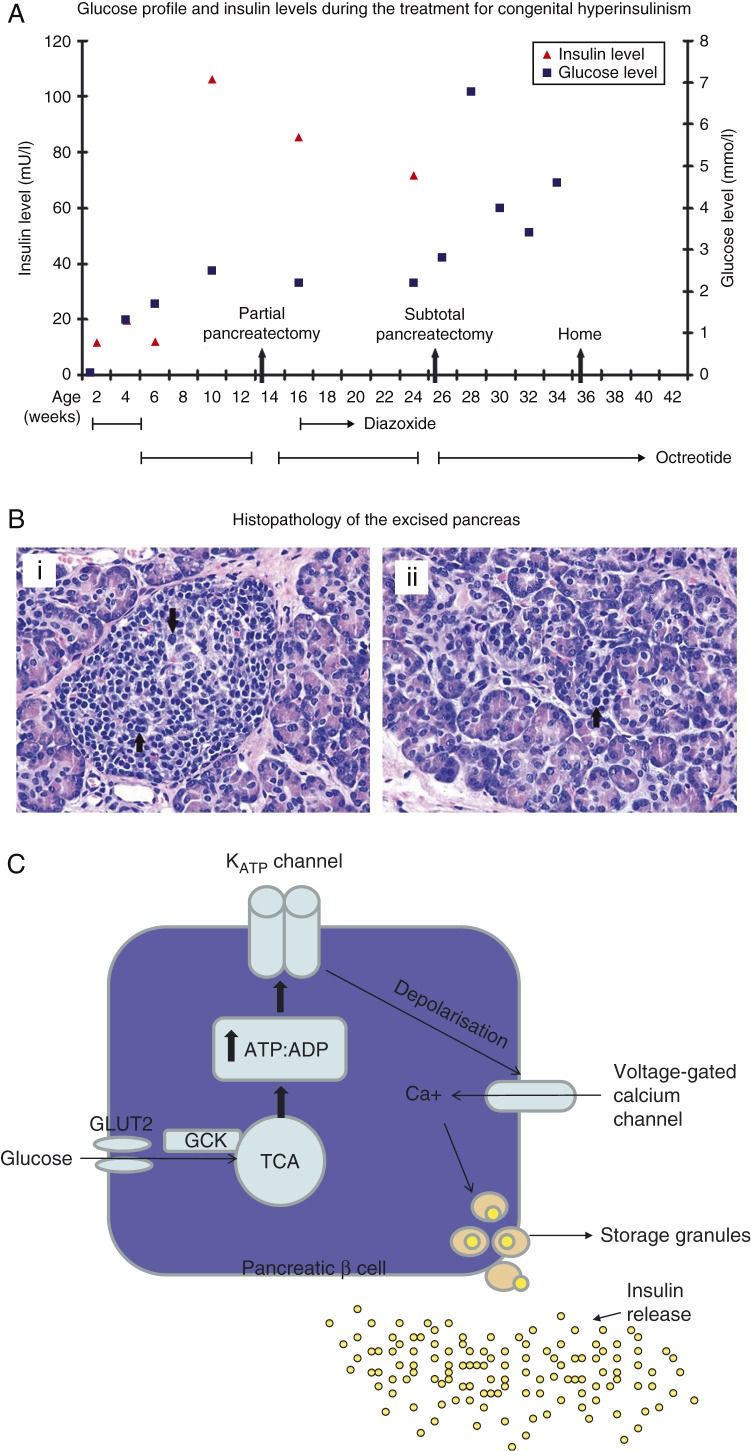
(A) Glucose and insulin profile during the treatment period. Medical and surgical treatments are plotted against the age of the patient. (B, i) Haematoxylin- and eosin-stained photomicrograph (magnification ×400). This shows a large islet with scattered enlarged and hyperchromatic nuclei (arrows). (B, ii) Haematoxylin- and eosin-stained photomicrograph (magnification ×400). This islet shows an endocrine cell with enlarged and hyperchromatic nucleus (arrow). (C) Pancreatic β-cell: with entry of glucose into the cell, an increase in ATP:ADP ratio occurs and this in turn close the K_ATP_ channel, resulting in depolarisation of the cell, allowing influx of calcium and finally exocytosis of vesicles leading to release of insulin.

A term male infant was born to non-consanguineous Asian parents by caesarean section. Maternal antenatal screen was unremarkable. His birth weight was 3230 g (50th–90th percentile) and length and head circumference were gestational age-appropriate. Apgar scores at birth were 9 at 1 min and 10 at 5 min. He was non-dysmorphic and systemic examination was unremarkable. At 3 h of life, he was noted to be lethargic and on checking his blood glucose level it was unrecordable. He was treated with i.v. glucose infusion and s.c. glucagon but remained hypoglycaemic even on a GIR of 12.6 mg/kg per min. During an episode of hypoglycaemia (1.2 mmol/l), his insulin level was 11.7 mU/l (Fig. 1). The serum growth hormone, lactate, ammonia and acylcarnitine levels were normal. His blood glucose levels normalised with a GIR of 16.4 mg/kg per min.

When feeds were commenced, hypoglycaemia recurred. The patient was started on diazoxide (with hydrochlorothiazide) at 10 mg/kg per day on day 3 and then increased to 20 mg/kg per day but continued to have persistent hypoglycaemia. As the patient failed to respond to diazoxide, s.c. octreotide was started at the dose of 10 μg/kg per day. His blood glucose profile remained labile and hence the dose of octreotide was increased to 20 μg/kg per day. As the patient was medically unresponsive to diazoxide, genetic studies were undertaken for the cause of HH.

## Investigation

Genetic studies were done in the molecular genetics laboratory in Exeter, UK. Sequence analysis for the *ABCC8* gene showed a paternally inherited heterozygous missense mutation (p.D1472N; c.4414G>A) and failed to detect a change from the normal sequence in the *KCNJ11* gene. A second *ABCC8* mutation of maternal origin was not found by sequence or dosage analysis multiplex ligation-dependent probe amplification (MLPA). The sequence analysis included the coding regions, conserved splice sites and a recently reported deep intronic splicing mutation (1). This paternally inherited *ABCC8* (*p.D1472N*) mutation was previously reported in a patient with a focal (tail) pancreatic lesion (2). As ^18^F-DOPA-PET/CT was unavailable locally in our country, ^68^G–DOTATATE-PET nuclear scan was done but the results were non-conclusive. A pre-operative high-resolution ultrasound scanning was attempted and a questionable small nodular focus, which distorted the anterior contour at the junction of the pancreatic body and medial tail region, was identified.

## Treatment

The patient failed to respond to diazoxide (up to 20 mg/kg per day) and his blood glucose levels could only be kept stable on a high dose of s.c. octreotide infusion. He therefore underwent partial pancreatectomy (50%) on day 103 of life, leaving the head and part of the body of pancreas. Post-operatively, he continued to have persistent HH (Fig. 1A) and the GIR remained high at 17 mg/kg per min to maintain normoglycaemia despite being on 20 μg/kg per day octreotide. Histopathological examination of the resected pancreas showed absence of any focal lesions. The pancreas had normally sized (Fig. 1Bi) to large (Fig. 1Bii) islets. The islets showed scattered endocrine cells with large and hyperchromatic nuclei (Fig. 1Bi and ii). These changes are consistent with typical diffuse disease. Owing to the persistent hypoglycaemia, he subsequently underwent a subtotal pancreatectomy (95%) on day 183 of life. In the immediate post-operative period, he required a GIR of 10 mg/kg per min to maintain normal blood glucose levels, but over the next 2 weeks, his blood glucose levels stabilised.

## Outcome and follow-up

The patient required nasogastric tube feeding due to poor oro-pharyngeal coordination and he achieved full enteral feeds by the 30th post-operative day. Gastro-oesophageal reflux disease was treated with anti-reflux medication. He was discharged on day 244 of life with pre-feed home blood glucose monitoring and once-daily injections of octreotide (10 μg/kg per day), which was later discontinued on day 301 of life. His growth and neuro-developmental assessment at the age of 1 year was normal.

## Discussion

HH is the commonest cause of persistent and recurrent hypoglycaemia during the neonatal and infancy periods. Insulin inhibits gluconeogenesis and glycogenolysis and stimulates uptake of glucose in muscles and adipocytes, leading to hypoglycaemia. Moreover, insulin inhibits lipolysis and thereby ketone body synthesis. In effect, the brain of the baby with CHI is deprived of the primary (glucose) and secondary (ketones) energy sources due to inappropriately elevated insulin levels.

CHI is a genetically heterogeneous disease characterised by dysregulated insulin secretion from pancreatic β-cells (Fig. 1C). In the face of hypoglycaemia, infants with CHI have inappropriately elevated serum insulin, low ketone bodies, low fatty acids and show a glycaemic response to glucagon. Infants with CHI typically need a GIR of more than 8 mg/kg per min to maintain normoglycaemia (3). The incidence of sporadic forms of CHI is estimated at 1 in 40 000 live births, but in familial forms, it may be as high as 1 in 2500 with substantial consanguinity (3).

CHI has a strong genetic basis and mutations in the key genes (*ABCC8*, *KCNJ11*, *GLUD1*, *GCK*, *HADH*, *SLC16A1*, *HNF4A*, *HNF1A* and *UCP2*) regulating insulin secretion have been identified. Integrity of the pancreatic β-cell ATP-sensitive potassium (K_ATP_) channel depends on the interactions between the pore-forming inward rectifier potassium channel subunit (KIR6.2) and the regulatory subunit sulfonylurea receptor 1 (SUR1). The *ABCC8* and *KCJN11* genes (both localised to chromosome 11p15.1) encode the two components of K_ATP_ channel and most of the severe forms of CHI are due to recessively inactivating mutations of these genes. CHI can be focal, diffuse or atypical but clinically indistinguishable (4).

CHI with a paternally inherited heterozygous mutation suggested focal disease as described by Hardy *et al*. (2). They reported a case of diazoxide-unresponsive CHI with the *ABCC8/p.D1472N* gene mutation and loss of heterozygosity (LOH), having focal CHI on ^18^F-DOPA-PET/CT study, which was cured by focal excision. As LOH is necessary in the pathogenesis of focal CHI, the LOH status by microsatellite study is important in infants with paternally inherited heterozygous *ABCC8* mutations. In a study involving 36 Japanese infants with CHI, 84% had paternally inherited monoallelic mutations that accurately predicted the presence of the focal forms (5). Flanagan *et al*. (6) reported 150 homozygous, compound heterozygous and heterozygous inactivating mutations in *ABCC8* gene and 24 mutations in the *KCNJ11* gene. In a recent report of 300 patients with CHI, mutations were identified in only 45% of the patients, 36% being mutations in either *ABCC8* or *KCNJ11* gene. The focal forms seen in 40–50% of CHI cases occur due to the inheritance of paternal *ABCC8/KCJN11* mutations and a somatic loss of the maternal chromosome 11p15 region with a paternal isodisomy at the same locus (7). A report on the molecular spectrum of 109 diazoxide-unresponsive patients with *ABCC8* and *KCNJ11* gene mutations concluded that in patients with single K_ATP_ channel mutations, ^18^F-DOPA-PET/CT imaging to differentiate focal and diffuse forms should follow genetic analysis (8).

In our particular case, the genetic analysis showed a paternally inherited *ABCC8/p.D1472N* mutation, predictive of focal disease. He underwent partial pancreatectomy on the basis of the genetic report and the suggestion of focal pancreatic lesion in the intra-operative high-resolution ultrasound scan. Imaging using ^18^F-DOPA-PET/CT is locally unavailable. Histopathology of the excised pancreas showed diffuse disease instead and the baby remained hypoglycaemic post-operatively. CHI due to large focal lesions can be excluded by histopathology and microsatellite analysis for LOH for maternal allele in the pancreatic lesion, the latter could not be done in our case. Autosomal dominant inheritance of CHI is unlikely given the previous case with a focal lesion (2) but cannot be ruled out. Dominant *ABCC8* mutations typically cause diazoxide-responsive disease (3), but Flanagan *et al*. (9) reported dominant *ABCC8* mutations in five patients from four families who were unresponsive to diazoxide and were cured by near total pancreatectomy. It is also possible that a second non-*ABCC8*/non-*KCNJ11* mutation may be present causing the diffuse disease, as nearly 87% of diazoxide-unresponsive infants have either the *ABCC8* or *KCNJ11* mutation with the genetic cause of the remainder still unknown (7). The most likely explanation is that a maternally inherited *ABCC8* mutation is present in a regulatory or intronic region of the gene but has not been detected by Sanger sequencing or dosage analysis. Next-generation sequencing provides a means to analyse the entire genomic region rather than just the coding exons and conserved splice sites (1) and is the logical next test.

A precise pre-operative assessment of CHI is now possible since the inception of ^18^F-DOPA-PET/CT scan, the current gold standard imaging technique having high specificity (100%) and sensitivity (88–94%) for focal lesions (2). The ^68^G–DOTATATE-PET nuclear scan used in our case is indicated primarily for the diagnosis of neuroendocrine tumours (10) and there is limited information on the role of high-resolution intraoperative ultrasound of the pancreas in CHI. This case demonstrates that ^18^F-DOPA-PET/CT imaging to confirm focal disease is essential in the management of infants with diazoxide-unresponsive CHI and a paternally inherited heterozygous mutation.

Diazoxide is the drug of choice for medical therapy once HH is confirmed (3). Diazoxide prevents β-cell membrane depolarisation and inhibits insulin secretion by keeping K_ATP_ channels open in patients who are diazoxide responsive, thereby allowing medical management without the need for pancreatectomy. Fluid retention and hypertrichosis are common with diazoxide use (3). Hypertrichosis was noted in the reported case while on diazoxide, which subsequently resolved on discontinuation. Most centres use chlorothiazide in conjunction with diazoxide to counteract the potential fluid retention side effects. Diazoxide is not useful in diffuse forms of CHI due to inactivating mutations in *ABCC8* and *KCNJ11* genes and in focal forms (3). If hypoglycaemia is unresponsive to diazoxide, a rapid genetic analysis for common mutations followed by imaging if indicated, facilitates differentiation of focal and diffuse forms in majority of patients with CHI. Laparoscopic focal excision is curative in focal forms whereas subtotal pancreatectomy leads to diabetes and exocrine pancreatic deficiency in most of the infants with diffuse disease (11).

The infants on treatment for CHI should have long-term developmental and visual follow-up because of the high risk of neurodevelopmental delay, cerebral palsy and epilepsy following hypoglycaemia-induced brain injury (12) (13).

Management of CHI is challenging even in developed countries due to lack of facilities for genetic studies and ^18^F-DOPA-PET/CT scan, currently limiting these diagnostic facilities only to specialised centres around the world. Diagnosis and treatment of our patient could have been expedited if these facilities were locally or regionally available.

## Patient consent

Written informed consent has been obtained from the father of the patient for publication of the submitted article and accompanying images.

## Author contribution statement

S Chandran recruited the patient, collected data and wrote the manuscript. F Yap Kok Peng was directly involved in the management of the patient throughout the hospital stay and currently the lead person in the follow-up program. V S Rajadurai discussed and finalised all decisions related to the management of the patient. Y Te Lu is surgeon who performed the partial and subtotal pancreatectomy for this reported case. K T E Chang reported the pancreatic histopathology. S Flanagan carried out the genetic testing for *KCNJ11* and *ABCC8* gene mutations. S Ellard supervised the genetic testing and edited the final version of the manuscript. K Hussain helped to write the manuscript and edited the final version.
